# A Narrative Review of Cerebellar Malfunctions and Sleep Disturbances

**DOI:** 10.3389/fnins.2021.590619

**Published:** 2021-06-25

**Authors:** Bijia Song, Jun-Chao Zhu

**Affiliations:** ^1^Department of Anesthesiology, Shengjing Hospital of China Medical University, Shenyang, China; ^2^Department of Anesthesiology, Beijing Friendship Hospital of Capital Medical University, Beijing, China

**Keywords:** cerebellum, sleep disturbances, sleep–wake cycle, REM sleep, NREM sleep, sleep–wakefulness transition

## Abstract

Cerebellar malfunctions significantly impact the regulation of the sleep–wakefulness transition. The possible mechanism for this effect is still unknown. Evidence on the role of cerebellar processing in the sleep–wake cycle is derived mainly from animal studies, and clinical management of the sleep–wake cycle is also challenging. The purpose of this review is to investigate the role of cerebellar activity during normal sleep and the association between cerebellar dysfunction and sleep disorders. Large-scale, multicenter trials are still needed to confirm these findings and provide early identification and intervention strategies to improve cerebellar function and the sleep quality of patients.

## Introduction

The cerebellum is often regarded as the “little brain” or “neuronal machine.” It is a critical component of the central nervous system that coordinates many brain functions according to the Marr–Albus model (Albus, 1971), such as motor planning (Gao et al., 2018), motor execution (Becker and Person, 2019), and motor learning (Lee et al., 2015). Moreover, cerebellar projections are also thought to influence respiration (Xu and Frazier, 2000) and cognition (Schmahmann and Sherman, 1997), as well as mediate the detection of sensory discrepancies (Blakemore et al., 2001) and prediction of sensory events (Nixon and Passingham, 2001). The spontaneous sleep–wake cycle is a recursive organization involving two distinctive states, namely, non-rapid eye movement (NREM) and rapid eye movement (REM) sleep. NREM sleep is further divisible into N1, N2, and N3 stages (Berry, 2015). A previous study on cerebelloctomized cats reported a small decrease in wakefulness and NREM sleep, and an increase in REM sleep, which revealed that the cerebellum may participate in fine-tuning and regulation of the sleep–wake cycle (Cunchillos and De Andrés, 1982). At present, the available evidence on the role of the sleep–wake cycle in cerebellar processing is mainly from animal studies (Barik and de Beaurepaire, 2005; Zhang et al., 2020), and to date the clinical management of the sleep–wake cycle is also challenging. This highlights an important area clinically, which can be further explored through future studies. Here, we summarize the current knowledge on cerebellar activity during the sleep–wake cycle and the relationship between the malfunctioning of the cerebellum and sleep disturbances. We also discuss the mechanisms of sleep disturbances and their adverse effects.

## The Relationship Between Cerebellar Activity and Sleep

### Cerebellar Activity During the Sleep–Wake Cycle

Sleep is an important brain function in humans, because it supports not only cognitive processes such as memory retrieval, learning, and attention but also language processing, decision making, and even creativity (Diekelmann and Born, 2010; Diekelmann, 2014). The normal sleep pattern can be subdivided into two stages, REM and NREM sleep that cyclically alternate during the night. Studies have found that the medial parabrachial nucleus (MPB), an important part of the parabrachial nucleus located in the surrounding region of the superior cerebellar peduncle (SCP), is involved in the transitioning of the sleep stage (Fuller et al., 2011; Anaclet et al., 2012), particularly switching of REM sleep to NREM sleep and vice versa (Hayashi et al., 2015). Marchesi and Strata (1970) also demonstrated that the sleep–wake cycle in humans reflected the intrinsic activity of cerebellar neurons and their afferents. “The activity of the afferents is related to/correlates with that of climbing fibers and mossy fibers, which separately originate from the inferior olive and a variety of other sources in the brain stem” (De Zeeuw et al., 2011). The potential mechanism of cerebellar activity during sleep may be due to the activity of climbing fibers eliciting complex spikes in the Purkinje cells, and mossy fibers regulating the Purkinje cells’ simple spike activity. Both climbing and mossy fibers show relatively low and high levels of activity during NREM and REM sleep, respectively (Marchesi and Strata, 1971), which reveal the possible existence of sleep-stage-dependent cerebellar activity.

### Cerebellar Activity During NREM Sleep

In the cerebral cortex, NREM sleep is divided into NREM 1–2 (lighter sleep stages) and deep slow-wave sleep (SWS). K-complexes are single slow waves typically occurring during NREM stage 2, while more continuous slow waves occur in what is considered deep sleep or NREM stage 3 (Riedner et al., 2011). In the cerebellum, local field potentials have been recorded during NREM sleep. Cerebellar signals during NREM stage 1 are lower compared with those during wakefulness (Diedrichsen et al., 2010); cerebellar functional magnetic resonance imaging (fMRI) signals during NREM stage 2 co-occur with K-complexes (Jahnke et al., 2012) and sleep spindles (Schabus et al., 2007), while cerebellar fMRI signals during NREM stage 3 co-occur with slow waves at the neocortex (Kaufmann et al., 2006). Moreover, since cerebellar fMRI signals largely reflect mossy and parallel fiber activity (Kaufmann et al., 2006), it was reported that mossy fibers derived from the pons contribute to cerebellar NREM sleep stages by decreasing their excitatory drive. Meanwhile, the results of positron emission tomography (PET) studies combined with electroencephalography recordings also indicated decreased cerebellar activity during the transition from pre-sleep wakefulness to SWS (Kjaer et al., 2002; Hiroki et al., 2005).

### Cerebellar Activity During REM Sleep

In humans, REM sleep accounts for 20–25% of total sleep, which is characterized by θ activity, although this is not continuous (Wichniak et al., 2017). The cerebellum participates in the production of REM atonia and phasic activity of the lateral rectus muscles of the eyes (Gadea-Ciria and Fuentes, 1976). In contrast to NREM sleep, both the hemispheres and vermis of the cerebellum show increased activity during REM sleep, which may indicate increased activity in their mossy fiber-parallel fiber pathways during this sleep stage (Braun et al., 1997; Hong et al., 2009; Miyauchi et al., 2009). Previous animal studies also reported that the activity of the Purkinje cells and that of neurons of the cerebellar nuclei both increase during REM sleep, with the neurons in the fastigial nucleus showing the strongest trends (Hobson and McCarley, 1972; Palmer, 1979). In addition, it has been shown that the cerebellum regulates autonomic inputs from the amygdala, periaqueductal gray (PAG), and thalamus and expresses parasympathetic and sympathetic outputs to the brainstem ventilatory and oculomotor neurons during REM sleep (Dharani, 2005).

### Role of Cerebellar Malfunctions in Sleep Disturbances

In humans, malfunction of the cerebellum not only impairs motor control and motor memory formation (Krakauer and Shadmehr, 2006; De Andrés et al., 2011; De Zeeuw et al., 2011; Gao et al., 2012) but also may lead to changes in the sleep–wake cycle (Pedroso et al., 2011) and even cause sleep disorders (insomnia, excessive daytime sleepiness, REM behavior disorder, and sleep apnea) (Pedroso et al., 2011; DelRosso and Hoque, 2014; Canto et al., 2017). Studies have found that patients with spinocerebellar ataxias showed increased daytime somnolence as well as NREM- and REM-related parasomnias (Silva et al., 2016; Martinez et al., 2017). In addition, the inhibitory activity of REM-OFF neurons in the locus coeruleus (LC) is a prerequisite for REM sleep. Thus, it is believed that increased CO_2_ levels during stable NREM sleep may hyperpolarize LC neurons, including REM-OFF, which may help initiate REM sleep (Madan and Jha, 2012). However, Liu et al.’s (2020) study indicated that in the cerebellar ataxia mouse model, variability in internal respiratory rhythms was reduced compared with that of healthy mice. This may lead to abnormal levels of physiological CO_2_, which consequently leads to sleep disturbances (Liu et al., 2020). Moreover, cats with lesions in the cerebellar vermis and hemispheres (De Andrés et al., 2011) and cerebellectomized cats (Cunchillos and De Andrés, 1982) showed abnormal sleep–wake cycles, which were characterized by an increased mean duration of NREM and total duration of REM periods and decreased mean number of sleep periods during the sleep–wake cycle (De Andrés and Reinoso-Suàrez, 1979). Further, lesions of the superior peduncle in cats resulted in a decreased mean duration (De Zeeuw et al., 2011) and total time of NREM and REM sleep (Cunchillos and De Andrés, 1982). The possible mechanism by which the cerebellum regulates sleep–wake cycles may be as follows: electrical stimulation on the cerebellar fastigial nucleus may relieve sleep disturbances by promoting the synthesis of 5-hydroxytryptamine in the cerebral cortex, hippocampus, and hypothalamus, and regulating norepinephrine levels in the frontal lobe and the hypocretin energy system in the lateral and dorsal regions of the hypothalamus (Wang et al., 2014). Moreover, using an adeno-associated viral vector (serotype rh10), Hashimoto et al. (2018) found that lobules VIII and IX of the rat cerebellar vermis directly projected their axons to the MPB. In addition, retrograde labeling of the MPB confirmed that the Purkinje cells of lobules VIII–X directly projected their axons to the ipsilateral MPB. These findings suggested that lobules VIII–X may regulate the neural activity of the MPB to modulate sleep–wake cycles (Hashimoto et al., 2018). Furthermore, the LC, ventrolateral region of PAG, and paraventricular hypothalamic nucleus (PVN) project their axons to lobule IX of the cerebellar vermis as precerebellar nuclei. The LC and PVN are involved in modulating the sleep–wake cycle (Tsujino and Sakurai, 2013). Further, the ventrolateral part of the PAG contributes to the negative regulation of REM sleep (Sapin et al., 2009). Considering this, lobule IX of the cerebellar vermis appears to play an important role in the neural circuit for modulating the sleep–wake cycle through the MPB. However, whether lobules VIII and X have the same neural circuit as lobule IX still needs to be confirmed by further studies. Other possible mechanisms by which cerebellar malfunction causes sleep disturbances may include the following. It has been reported that the medial preoptic anterior hypothalamus has heat-sensitive neurons, which are necessary for sleep initiation and maintenance (Saper et al., 2001). It has also been reported that the neurons promoting awakening and sleep could inhibit each other, resulting in stable awakening and sleep. The cerebellum and hypothalamus are interconnected and control autonomic regulation and emotion through histaminergic (Dietrichs and Haines, 1989) and cold sensitive neurons and ultimately maintain the sleep cycle within the physiological range (Mallick and Alam, 1992; Jha and Mallick, 2009). Some research also indicates that the link between the hypothalamus and cerebellum is through the SCP and middle cerebellar peduncle (MCP), not the inferior cerebellar peduncle. Compared to the MCP, the SCP has more extensive connections. The hypothalamic connection through the SCP and MCP is denser than the cerebellar–hypothalamic connection. Therefore, the effect of the hypothalamus on the cerebellum is more prominent than that of the cerebellum on the hypothalamus. In addition, bidirectional connections between the lateral hypothalamic nucleus and the cerebellum *via* the SCP and ventromedial hypothalamic nucleus, as well as bidirectional connections to the cerebellum *via* the MCP, may represent circuits in which the cerebellum may be subject to and affect higher emotions and autonomic centers (Çavdar et al., 2018). This interaction may also explain how sleep disorders affect cerebellar function.

### Role of Sleep Disturbances in Cerebellar Malfunction

Patients with cerebellar malfunction experience sleep disturbance, and patients with primary sleep disturbances such as chronic insomnia, fatal familial insomnia, or obstructive sleep apnea (OSA) accompanied by daytime sleepiness (Macey et al., 2008) also show decreased cerebellar volume and cerebellar malfunction (Desseilles et al., 2008). For example, patients with REM sleep behavior disturbances reported a volumetric decrease in the anterior lobes of the cerebellar cortex and cerebellar nuclei (Boucetta et al., 2016). In addition, under normal physiological conditions and the regulation of relevant neurotransmitters secreted by the central nervous system, the sleep and awakening states are relatively balanced. Gamma aminobutyric acid (GABA) is one of the most abundant neurotransmitters in the brain, which has an inhibitory effect on brain activity (Pérez-Rico et al., 2014; Ben Saad et al., 2015). Glutamate is also an important neurotransmitter, which acts as a stimulant for brain activity. Thus, the abnormal ratio of GABA to glutamate may adversely affect the regulation of the sleep–wake cycle (Kuczyński et al., 2016). OSA has been associated with sympathetic activation, excessive daytime sleepiness, and cognitive function changes. Xu et al.’s (2018) study concluded that OSA is closely related to metabolic disorders. In a chronic intermittent hypoxia animal model, the weight of the body, cerebellum, and hippocampus as well as glutamate levels in the cerebellum and hippocampus decreased (Xu et al., 2018). Moreover, Yadav et al. (2013) found that patients with OSA had decreased blood flow in the SCPs, corticospinal tracts, pontocerebellar fibers, and midbrain red nucleus. These areas were potentially damaged due to cytotoxicity secondary to intermittent hypoxia (Macey et al., 2008; Yadav et al., 2013). Patients with chronic insomnia usually show an increase in brain metabolism both during sleep and during wakefulness. Similarly, patients with fatal familial insomnia, an extremely rare autosomal-dominant prion disease characterized by insomnia, dysautonomia, and somatomotor abnormalities (for example, cerebellar ataxia and dysarthria), also demonstrate serious thalamic hypometabolism and apoptotic neurons in the thalamus and medullary olives by PET and neuropathologic data (Pedroso et al., 2013). With time, patients with fatal familial insomnia develop moderate atrophy of the cerebellum, with spongiform changes in the cerebellar cortex (Cortelli et al., 2014). The mechanism, although still unclear, may be secondary to intrinsic cerebellar dysfunction.

## The Adverse Effects of Sleep Disturbances

### The Relationship of Sleep Disturbances With Alzheimer’s Disease and Dementia

In recent years, there has been increasing evidence of a relationship between sleep disorders and common neurological diseases, such as neurodegenerative diseases, dementia, Alzheimer’s disease, Parkinson’s disease, and depressive disorders. Studies have mainly focused on the association between OSA and cognitive function, dementia, and Alzheimer’s disease. OSA may increase the risk of mild cognitive impairment or dementia up to two to six times (Yaffe et al., 2011; Chang et al., 2013). In patients with sleep disorders, OSA may be the core mechanism leading to Alzheimer’s disease (Emamian et al., 2016). Specifically, chronic hypoxia increases Aβ plaque formation in cellular and mouse models (Li et al., 2009), whereas acute hypoxia promotes Tau phosphorylation (Fang et al., 2010). In adults with normal cognitive function, OSA is found to coincide with lower cerebrospinal fluid Aβ and Tau levels, thus pathologically associating hypoxia with Alzheimer’s disease. Sleep apnea may involve multiple damaging alterations in the brain, thereby leading to OSA and neurodegenerative diseases. MRI and diffusion tensor imaging measurements in patients with sleep apnea have shown a loss of regional volume and white matter integrity in the hippocampus, cingulate cortex, and cerebellar regions (Zimmerman and Aloia, 2006; Kumar et al., 2008; Macey et al., 2008; Joo et al., 2013; Kim et al., 2013). Disordered breathing during sleep potentially increases the risk of cerebrovascular disease. OSA is also a risk factor for stroke, bradycardia, supraventricular tachycardia, ventricular tachycardia, and atrial fibrillation (Arzt et al., 2005; Marin et al., 2005; Hermann and Bassetti, 2009), all of which are risk factors of dementia.

### The Relationship of Sleep Disturbances With Parkinson’s Disease

Besides new evidence that has identified sleep quality as a potential important risk factor for the development and progression of Alzheimer’s disease (Irwin and Vitiello, 2019; Leng et al., 2019), research also indicates that sleep disorders increase the risk of the development of Parkinson’s disease. A large, retrospective cohort study of more than 91,000 participants in Taiwan with non-apnea sleep disorders and no evidence of Parkinson’s disease found that sleep disturbance was an independent risk factor for Parkinson’s disease in comparison to matched controls who did not report sleep disturbances (Hsiao et al., 2017). It is worth mentioning that patients with chronic insomnia (lasting for more than 3 months) were mostly at risk. A longitudinal study of shift nurses also revealed an increased risk of Parkinson’s disease compared to nurses who did not work night shifts, illustrating that sleep abnormalities have a significant disease-modifying effect (Postuma et al., 2012; Barber et al., 2018). Therefore, abnormal sleep is considered to be intricately related with the risk, prodrome, and symptomatic progression of Parkinson’s disease (Bohnena and Hu, 2019).

Other studies have shown that daytime sleepiness and nighttime sleep problems appear to relate with fatigue, depression, urinary tract infections, cardiovascular disease, and dopamine agonist therapy. These findings support the association of sleep disturbance with Parkinson’s disease and the exacerbation of Parkinson’s disease symptoms, which is also strongly related to the patients’ quality of life and potential prognosis (Kurtis et al., 2013). In addition, sleep disturbances have been linked to cortical thinning, a hallmark of cortical atrophy found in many dementia subtypes (Möller et al., 2016). In elderly adults without cognitive impairment, sleep duration of ≤6 h or ≥8 h (that is, short or long sleep duration) was associated with faster thinning of the frontotemporal cortex (Lo et al., 2014). Further reduction of the sleep duration may lead to degeneration of the hippocampus through a variety of pathways, including altered neuronal excitability, decreased synaptic plasticity, and decreased neurogenesis.

### The Relationship of Sleep Disturbances With Depressive Disorder

Many longitudinal studies have identified insomnia as an independent risk factor for developing or recurrent depression in young, middle-aged, and elderly adults (Ariel, 2010). Sleep deprivation is associated with the incidence of depressive disorder and mortality in the elderly compared with patients without sleep difficulties. Patients with sleep difficulties have reported a decline in the quality of life and increased symptoms of depression and anxiety (Crenshaw and Edinger, 1999). These elderly patients have also shown slower reaction speeds and greater cognitive dysfunction, such as impaired memory. Moreover, these patients have demonstrated balance, vision, and walking difficulties, despite receiving medications (Brassington et al., 2000). All these difficulties can increase the risk of falls. Studies have confirmed that low sleep efficiency, increased sleep latency, and reduced total sleep time are associated with a higher risk of death (Neikrug and Ancoli-Israel, 2010). Therefore, additional attention is required for patients with sleep disorders, to improve their sleep quality and quality of life.

## Conclusion

In this review, we discussed how the cerebellum is actively involved in regulating the sleep–wakefulness transition and the interactive relationship between cerebellar malfunction and sleep disturbances. Sleep disorders are also related to common neurological disorders, such as neurodegenerative diseases, dementia, Alzheimer’s disease, Parkinson’s disease, and depressive disorder. It is important to identify patients with preexisting cerebellar malfunctions, as they are at a higher risk of experiencing sleep disturbances. Large-scale, multicenter trials are still needed to confirm these findings and provide early identification and intervention strategies to improve cerebellar function and the sleep quality of patients.

## Author Contributions

Both authors contributed to drafting or revising of the article, gave final approval of the version to be published, and agreed to be accountable for all aspects of the work.

## Conflict of Interest

The authors declare that the research was conducted in the absence of any commercial or financial relationships that could be construed as a potential conflict of interest.
